# ^13^C labeling unravels carbon dynamics in banana between mother plant, sucker and corm under drought stress

**DOI:** 10.3389/fpls.2023.1141682

**Published:** 2023-05-08

**Authors:** Mathilde Vantyghem, Eline Beelen, Rebecca Hood-Nowotny, Roel Merckx, Gerd Dercon

**Affiliations:** ^1^ Soil and Water Management & Crop Nutrition Laboratory, Joint FAO/IAEA Centre of Nuclear Techniques in Food and Agriculture, Department of Nuclear Sciences and Applications, International Atomic Energy Agency, Vienna, Austria; ^2^ Division of Soil and Water Management, Department of Earth and Environmental Sciences, KU Leuven, Heverlee, Belgium; ^3^ Institute of Soil Research, Department of Forest and Soil Sciences, University of Natural Resources and Life Sciences Vienna, Vienna, Austria

**Keywords:** carbon allocation, isotopic labeling, Musa, source-sink, water stress, phloem sap, CORM

## Abstract

Banana is a perennial crop and typically consists of a mother plant and one or more suckers that will serve as the next generation. Suckers are photosynthetically active, but also receive photo-assimilates from the mother plant. While drought stress is the most important abiotic constraint to banana cultivation, its effect on suckers or banana mats as a whole remains unknown. To investigate whether parental support to suckers is altered under drought stress and to determine the photosynthetic cost to the parental plant, we conducted a ^13^C labeling experiment. We labeled banana mother plants with ^13^CO_2_ and traced the label up to two weeks after labeling. This was done under optimal and drought-stressed conditions in plants with and without suckers. We retrieved label in the phloem sap of the corm and sucker as soon as 24 hours after labeling. Overall, 3.1 ± 0.7% of label assimilated by the mother plant ended up in the sucker. Allocation to the sucker seemed to be reduced under drought stress. The absence of a sucker did not enhance the growth of the mother plant; instead, plants without suckers had higher respiratory losses. Furthermore, 5.8 ± 0.4% of the label was allocated to the corm. Sucker presence and drought stress each led to an increase in starch accumulation in the corm, but when both stress and a sucker were present, the amount was severely reduced. Furthermore, the second to fifth fully open leaves were the most important source of photo-assimilates in the plant, but the two younger developing leaves assimilated the same amount of carbon as the four active leaves combined. They exported and imported photo-assimilates simultaneously, hence acting as both source and sink. ^13^C labeling has allowed us to quantify source and sink strengths of different plant parts, as well as the carbon fluxes between them. We conclude that drought stress and sucker presence, respectively causing a reduction in supply and an increase in carbon demand, both increased the relative amount of carbon allocated to storage tissues. Their combination, however, led to insufficient availability of assimilates and hence a reduced investment in long-term storage and sucker growth.

## Introduction

Banana (*Musa* spp.) is the most important fruit globally (150 million t y^-1^) and serves both as staple food source and commercial crop (FAO, 2021). It is a perennial plant that propagates through the formation of suckers, also called daughter plants. Suckers develop from lateral buds on the underground corm. Their first leaves are narrow and have limited photosynthetic capacity. During this early stage, the suckers rely almost completely on carbohydrates from the mother plant (Eckstein and Robinson, 1999). Later, they develop normal leaves and become self-supportive. However, even when photosynthetically active, daughter plants continue to partly rely on the mother plant and compete for photo-assimilates with the developing bunch (Turner, 1972; Eckstein and Robinson, 1999; Dens et al., 2008; Mahdi et al., 2014; Dorel et al., 2016). This integrated system of mother plant, corm and daughter plant(s) is called a banana mat.

Drought stress is the most important abiotic constraint to banana production worldwide (Turner, 1995). In East-Africa, which has the highest banana consumption per capita, water unavailability is identified as the number one factor limiting yields (Carr, 2009; Van Asten et al., 2010; Wairegi et al., 2010; Adhikari et al., 2015; Gambart et al., 2020). A substantial body of research has been undertaken on the (physiological) effects of drought stress on banana, but the focus is in most cases on vegetative banana plants (Souza Santos et al., 2018; van Wesemael et al., 2019; Eyland et al., 2022). The effect of drought stress on suckers or on the functioning of the banana mat remains unknown.

Vantyghem et al. (2022) found that stable carbon isotope values (δ^13^C) in the leaves of suckers were lower (more negative) than in the leaves of the mother plants. δ^13^C values in leaf tissues are related to stomatal opening and can be used as an indicator for drought stress (Farquhar et al., 1982). The lower δ^13^C values in daughter plants, compared to mother plants, are likely a consequence of their sheltered position below the canopy, resulting in less stress. However, the fact that daughter plants partly rely on the mother plant for photo-assimilates, complicates the interpretation of their δ^13^C value. Moreover, it is possible that drought stress alters the carbon flux between mother and daughter plants. Hence, as of yet, we cannot interpret the δ^13^C values of daughter plants unambiguously and therefore, cannot know with certainty how much a daughter plant suffers from drought stress in comparison to the mother plant.

Carbon fluxes in banana mats have never been quantified. Experiments with source-sink manipulations (i.e. removing sources/sinks or severing their connection) have indicated that the bunch is the main sink for carbon assimilates, followed by the sucker (Eckstein et al., 1995; Dens et al., 2008). Before flowering, the growing leaves of the mother plant form an important sink as well and a significant portion of the assimilates is allocated to the pseudostem (the leaf sheaths) and the corm, which both serve as storage tissue (Eckstein et al., 1995). The corm and pseudostem become important sources of carbohydrates for the bunch later on and once the mother plant dies, for the sucker (Dens et al., 2008; Turner et al., 2020). The youngest seven to five leaves are the main source in the plant and the second youngest leaf has the highest photosynthetic activity (Devos, 1984; Eckstein and Robinson, 1995; Thomas and Turner, 1998). Photo-assimilates are redistributed through the phloem in the vascular bundles. In banana, all leaves develop from the underground corm. Consequently, carbohydrates being transported from source to sink leaves or from mother to daughter plant must pass through the corm. The corm thus fulfills a dual role as transport and storage organ.

The quantity and speed of carbohydrate transport from source to sink in banana are currently unknown. The effect of drought stress on carbon dynamics and allocation, in particular to the sucker, has not been studied either. The main objective of this study was therefore to trace and quantify carbon fluxes in banana mats under optimal and drought conditions and in plants with and without suckers. This was done by labeling mother plants with ^13^CO_2_. We examined enrichment levels after labeling at fixed time intervals in source and sink tissues, as well as in the phloem sap. We hypothesized that drought would lead to a preferential carbon allocation to the growing leaves of the mother plant, at the expense of other sinks, such as the corm and daughter plant. Furthermore, as a second objective, we aimed to deepen our understanding of carbon allocation within certain sinks and sources. More specifically, we wanted to assess long-term storage in the corm and determine the usage of translocated carbon in leaves. For this purpose, we examined the enrichment levels of specific carbon compounds extracted from the corm and leaves.

## Material and methods

### Experimental set-up

The carbon-13 labeling experiment took place in the greenhouses of the Soil and Water Management and Crop Nutrition Laboratory of the Joint FAO/IAEA Centre of Nuclear Techniques in Food and Agriculture in Seibersdorf, Austria, between April and September 2021. Twenty-four young Grand Nain banana plants were obtained from the FAO/IAEA Plant Breeding and Genetics Laboratory and re-potted in 7 L pots filled with a 2:1 (v/v) mixture of peat and compost-based substrate (COMPO SANA^®^) and sand. The Grand Nain variety is known to have suckers that are fairly dependent on the mother plant (Dorel et al., 2016). After three months, the plants were re-potted in 21 L pots, using the same substrate. After five months, all plants had developed one or more suckers and reached an average pseudostem height of 53 ± 8 cm. A single sucker per plant was retained, as is the common practice in banana cultivation. The plants were distributed over six tables. Plants were watered three times per week and fertilized once per week with Substral^®^ 6:3:6 liquid fertilizer. This was supplemented with granular K_2_O which was supplied on two occasions. Due to significant spider mite problems, a vaporizer was employed to maintain the air humidity in the greenhouse at 60%.

Three weeks prior to ^13^C labeling, the treatments were initiated. The remaining one sucker was removed from half the plants, resulting in plants with (MD) and without (M) a daughter plant. Half the plants received optimal watering, while the other half underwent a drought treatment. The optimal treatment consisted of watering to 100% field capacity (100FC, gravimetric water content of 0.77 g.g^-1^), while the drought stress was induced by watering to 50% field capacity (50FC). Field capacity was determined by saturating four pots containing the substrate used for the experiment and allowing them to drain on a raster, while keeping the upper surface covered. The water content at which no more water drained from the pots, was considered to be field capacity. Plants were watered up to respectively 100FC or 50FC, by weight. Just before the start of the treatments, the weight of the plants (*m_plant_*) was approximated as


(1) $$m_{plant\;}=\;m_{total}-\;\left(m_{soil,\;dry}+\;m_{water}\;+\;m_{pot}\right)\;$$

where m_pot_ and *m_soil_* were known (with some uncertainty), *m_water_* was determined by taking a soil sample of each plant pot and gravimetrically determining its water content and *m_plant_* was assumed constant, hence plant growth was neglected, in line with standard practice.

With all masses known, a target weight was set for each individual plant pot, resulting in the appropriate treatment (50FC or 100FC). Plants were weighed and water levels adjusted daily accordingly. Leaf temperature was used as a measure for drought stress (Vantyghem et al., 2022). Measurements with a hand-held infrared thermometer were also done daily in the early afternoon, starting nine days after the initiation of the treatments. On sunny days (air temperature of 24 ± 1°C, n = 5) the average temperature of plants under the 100FC treatment was 32 ± 1°C, while the average for plants under 50FC was 35 ± 1°C. The difference between the treatments was significant on all non-clouded days.

### ^13^C labeling

The plants were labeled with ^13^CO_2_ in a 15 m^3^ growth chamber, eight pots at a time (resulting in three labeling groups). The growth chamber was kept at 25°C and a relative humidity of 60%. The chamber was equipped with LED growth lamps (Hillvert^®^ HT-Wedge 1200) with a photosynthetic flux density around 400 µmol m^-2^ s^-1^ (380-780 nm) at canopy level. For a detailed description of the growth chamber, we refer to Slaets et al. (2020). Just before labeling, all plants were watered to ensure sufficient uptake of the ^13^C label. The daughter plants and the pots were then sealed to prevent label uptake. This was done by enclosing them in 67 x 100 cm gas tight vacuum bags (IKEA^®^ Spantad). The bags were sealed with the double zip-locks in place and additionally with tape and flexible sealing clay (Teroson^®^ RB IX). Gas tightness of this system was tested in prior additional evaluations which are not described herein.

The CO_2_ pressure in the growth chamber was about 450 cm³.cm^-^³ when the plants were placed inside. To ensure sufficient label uptake, labeling was only initiated after the CO_2_ pressure had dropped to 250-300 cm³.cm^-^³, which took about two hours. The label was supplied by injecting 99% ^13^CO_2_ (2.3*10^6^ Pa, Sigma Aldrich^®^, St Louis, MO, USA) for 16 minutes at a rate of 400 ml.minute^-1^. This resulted in an ^13^C atom fraction in the growth chamber of approximately 96% and an estimated CO_2_ pressure of 9800 cm³.cm^-^³. The plants remained inside the growth chamber with enriched ^13^CO_2_ levels for an additional two hours.

The labeling was done on three different days, but always at the same time during the day (11am-1pm). A completely sealed control plant was added to each of the three labeling groups, to account for possible leakages of the bags. Moreover, six young untreated plants were labeled as well. These would later be kept in the greenhouse until they developed suckers (see section *3.4 Long term C dynamics*). After labeling, labeled plants were placed in a separate room of the greenhouse to avoid cross-contamination with unlabeled plants. δ^13^C levels in the room were monitored post-labeling through Off‐Axis Integrated‐Cavity Output Spectroscopy (Off‐Axis ICOS, Los Gatos Research^®^, San Jose, CA, USA) in flow through mode.

### Plant sampling

Samples of mother and daughter plants were collected just after labeling (0 h) and after 2, 4, 24, 48, 72 and 120 hours. Half of the plants (group A) were harvested after one week (168 h) and the other half (group B) after two weeks (336 h). Samples for natural abundance reference values were taken from four plants prior to labeling. The sealed control plants were sampled just before and after labeling.

**Figure 1:**
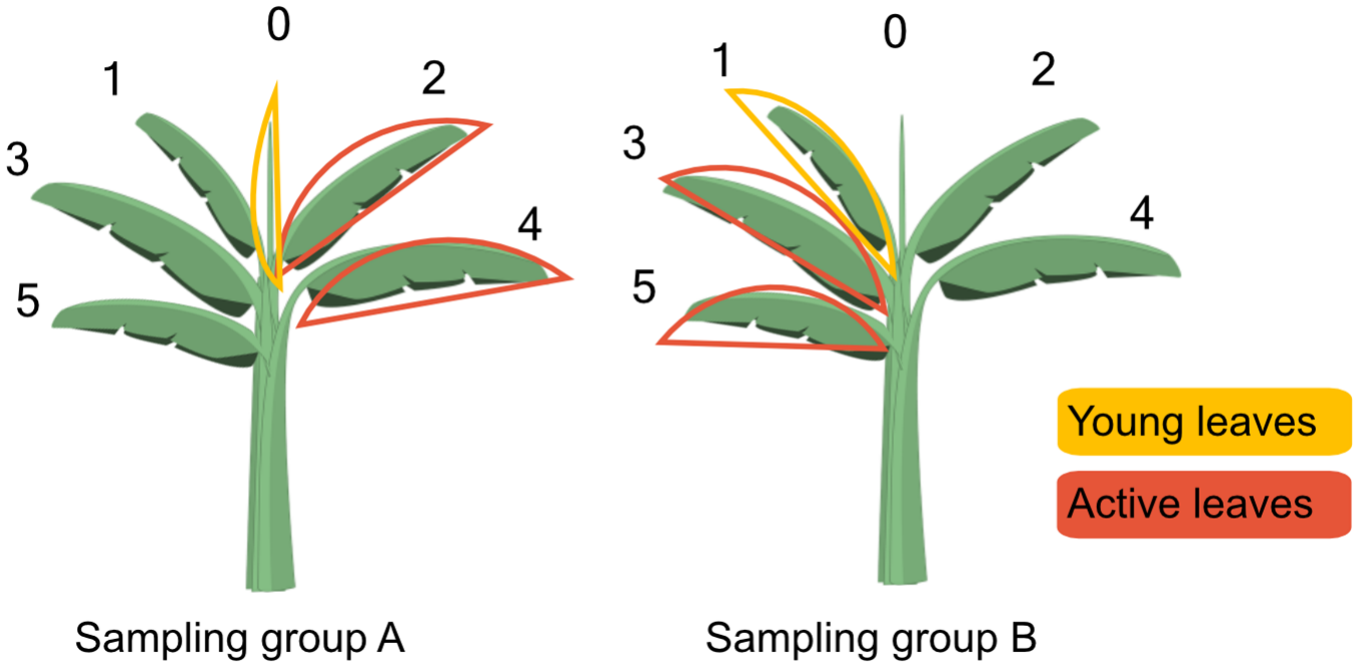
Scheme indicating the leaf sampling approach at 2, 4, 24, 48, 72 and 120 hours after labeling. In sampling group A, a sample of leaf 0 would serve as a sample of young leaves and a combined sample of leaves 2 and 4 as a sample of active leaves. In sampling group B, leaf 1 was sampled as a young leaf, while leaves 3 and 5 were sampled as active leaves. At the next sampling time, the selection would be reversed i.e. leaves 1, 3 and 5 would be sampled in group A and leaves 0, 2 and 4 in group B. This approach was taken to minimize the damage inflicted by repeated sampling. Plant design by Freepik.

Samples were collected from leaves, corm, and phloem sap, which was extracted from the leaf petioles and the corm. Leaf samples were taken from the young (partially developed) and active (fully developed) leaves (**Figure 1**). The young leaves were leaf 0 and 1. Leaf 0 was the still rolled up ‘cigar’ leaf and could only be sampled from the moment it partly unrolled. Leaf 1 was the youngest fully opened leaf. The active leaves consisted of leaf 2 to 5, with leaf 5 being the 5^th^ fully opened and oldest leaf. Just after labeling, samples were collected from every individual leaf in order to assess photosynthetic activity at leaf level and to obtain a correct estimate of overall label uptake. After that, each leaf was only sampled every other time to minimize the impact of repeated sampling. During a particular sampling event, leaves 0, 2 and 4 were sampled,while during the subsequent sampling event leaves 1, 3 and 5 were sampled. This selection was reversed for plants in groups A and B (**Figure 1**). Analysis was carried out on the groups of leaves (young and active), rather than on individual leaves. Leaf sampling generally consisted of puncturing a small disc (1 cm²) out of the lamina on both sides of the midrib. At 0h, 72h and 120h, however, entire leaf strips of about 5 cm width were taken to obtain enough biomass for compound extractions. Phloem sap sampling consisted of excising a 0.5 cm³ piece of petiole and placing it in an Eppendorf with 1 mL of Milli-Q water. This method does not exclude apoplast or xylem liquid, but the first mainly contains leaked carbon from the phloem sap and the second barely contains any carbon, hence, this method is considered appropriate for investigating the δ^13^C value of phloem sap.

Corm samples were collected by cutting a 1 cm³ piece from the upper part of the corm. The corm consists of a starchy central cylinder with a myriad of looping vascular bundles (Skutch, 1932). The central cylinder is surrounded by a cortex, and they are separated by a cambium layer. The vascular bundles originating from the source leaves are mostly concentrated around this cambium layer. In our plants, the cortex had a thickness of about 0.5-1 cm. Our samples thus included cortex, cambium, starchy parenchyma and vascular bundles. Half of the sample was kept for bulk analysis, while the other half was placed in 1 mL of water to extract the phloem sap (cf. leaf phloem sap).

At harvest, the plants were separated into leaves (laminae), petioles, corm and roots. Leaves and petioles were further divided into active, young and new leaves, the latter being the leaves that had appeared after labeling and all leaves still enclosed inside the pseudostem. All plant parts were accurately weighed. One corm was repeatedly sliced horizontally, and sub samples were taken from the resulting slices, by puncturing out small volumes, to make a rough assessment of the variability within the corm.

### Long-term C dynamics

To assess long-term storage of carbon in the corm and allocation to new suckers, the additional six young plants included in the labeling chamber were kept in the greenhouse until they developed suckers. Once a sucker appeared, it was removed from the main plant and prepared for analysis.

### Sample processing

Immediately after sampling, leaf, petiole and corm samples were microwaved for 5 minutes to stop biological activity. They were then oven-dried for 72 hours at 65°C. After drying, leaves and petioles were ground with a micro impact mill (Culatti^®^). Corm samples were ground in a mortar grinder (Retsch^®^ RM 200). For bulk analysis, 1-3 mg of ground material was weighed into a Sn capsule. Samples for sugar extraction were ground finer in a ball mill (Retsch^®^ MM 200). Leaf punches were weighed directly into Sn capsules.

Phloem samples (both from leaves and corm) were left to rest overnight at 5°C. Afterwards, the plant material was removed with tweezers and the watery samples were washed with heptane to remove latex-like components. In total 300 μL of the washed samples was pipetted into a Sn capsule, oven-dried and prepared for analysis.

Roots were initially kept within the soil and oven-dried for 72 hours at 50°C. Due to the organic nature of the substrate, washing the roots was deemed impossible. Instead, roots were sieved out of the dry soil. This method resulted in the inclusion of some soil and the organic material present in it. The initial substrate did not contain ^13^C enrichment, but there might be some enrichment introduced by root exudates and turnover. However, we consider this negligible compared to the much larger pool of natural abundance carbon in the substrate. The sieved roots were ground and prepared for analysis in the same way as the leaf and petiole samples.

### WSOM, cellulose and starch extraction

Water-soluble organic matter (WSOM) and cellulose were extracted from leaf samples. We adjusted the WSOM extraction method from Gessler et al. (2004). 50 mg of ground plant material in 1 ml of Milli-Q water was incubated at 5°C for 1 hour. Subsequently, the samples were heated to 100°C for 3 minutes to denature proteins and centrifuged (5 minutes at 12000 x g). Finally, 150 μL of the supernatant was transferred to a Sn capsule and oven-dried at 55°C.

The α-cellulose extraction method was conducted based on Leavitt and Danzer (1993). In short, sealed Teflon bags with 200 mg of ground leaf material were placed in an Erlenmeyer in a water bath at 70°C. In a first step lignin were removed through an acidified chlorite reaction. This step was repeated five times. After washing extensively, a 17% sodium hydroxide solution was added to the samples to remove hemi- and beta-cellulose. This step was repeated two more times. Finally, after washing again, the acidified chlorite reaction was repeated another two times. 1 mg of extracted cellulose was weighed into a Sn capsule and prepared for analysis.

Starch was extracted from corm samples according to Wanek et al. (2001). 50 mg of ground material was washed three times with a methanol-water-chloroform mixture (MCW, 12:5:3, v/v/v) to remove lipids. After drying, the samples were suspended in 0.5 mL Milli-Q water and left at 100°C for 15 minutes to gelatinize the starch. The gelatinized samples were then incubated in an α-amylase solution (1500 units mL^-1^) for 120 minutes at 85°C. Finally, the hydrolyzed starch was mixed with chloroform to denature and precipitate the α-amylase. 50 μL of the aqueous phase was pipetted into a Sn capsule and dried before analysis.

### Carbon isotope analysis and ^13^C_excess_ calculation

^13^C measurements were done with an elemental analyser – isotope ratio mass spectrometer (EA-IRMS). Most of the samples were analyzed in the FAO/IAEA Soil and Water Management & Crop Nutrition Laboratory in Seibersdorf, Austria (Vario Isotope Select, Elementar^®^, Langenselbold, Germany coupled to Isoprime 100, Elementar^®^), but bulk leaf samples and petioles were analyzed in the Soil and Water Management Laboratory of KU Leuven, Belgium (Thermo-Finnigan^®^ Delta V Advantage, Bremen, Germany).

The carbon isotope value was determined as follows


(2) $$\delta^{13}C\;\left(‰\right)=\frac{R_{sample}-\;R_{standard}}{R_{standard}}*1000$$

where *R* is the ratio between ^13^C and ^12^C and the standard being the Vienna-PDB standard (i.e. limestone from the Pee Dee formation in South Carolina). The atom fraction of ^13^C (*x(^13^C*)) was calculated as


(3) x(C13)=Rsample1+ Rsample=Rstandard(δ13C/1000+1)1+ Rstandard(δ13C/1000+1)

and the excess amount of ^13^C (*^13^C excess (g)*) as


(4) Cexcess13(g)=(x(13C)sample−x(13C)pre−labeling)·DWtissue·Ctissue100

where *x(^13^C)_pre-labeling_* is the ^13^C atom fraction before labeling, *DW* the dry weight (g) of the relevant plant tissue and *C* the percentage of Carbon. Pre-labeling values were obtained for bulk material from several plants in the experiment, as well as the control plants. All pre-labeling values are given in **Table 1**.

**Table 1:** Natural abundance δ^13^C values of different plant parts measured prior to labeling, which were used to calculate ^13^C_excess_.

Plant part	Fraction	Average	Sd	N
Leaves*	Bulk	*-24.78*	*0.79*	*4*
Leaves	WSOM	*-21.95*	*0.47*	*4*
Leaves	Phloem sap	*-27.23*	*1.23*	*3*
Corm	Bulk	*-25.44*	*1.65*	*7*
Corm	Phloem sap	*-25.44*	*1.65*	*7*
Roots	Bulk	*-26.34*	*0.02*	*4*

*Reference values for leaves obtained from laminae and used for both laminae and petioles.

Average, standard deviation (sd) and number of observations (N) are given.

For phloem sap samples, equation (4) was additionally multiplied with an extraction factor (mg WSOM/mg petiole). The extraction factor was determined by oven-drying and weighing petiole pieces after phloem sap extraction. The average extraction factor was 14.0 ± 4.1%. This value was also used for the corm phloem sap, which may have led to a slight overestimation.

Ultimately, excess values were expressed as a relative amount of ^13^C label i.e. as percentage of the initial label taken up by the plant:


(5) Cexcess13(%)=Cexcess,t13Cexcess,plant,t013

where *t* is the time of sampling (h) and *t_0_* the time just after labeling (0 h). *^13^C_excess,plant,t_0__* was determined as the sum of ^13^C_excess_ in the mother plant’s leaves, phloem sap and corm at t_0_. The initial label uptake of all plants is given in **Supplementary Table 1**. ^13^C_excess_ values mentioned in the text are relative ^13^C_excess_ values, unless explicitly indicated otherwise. Respiration losses of plants were calculated as the difference between the sum of ^13^C_excess_ in all plant parts at time of harvest and ^13^C_excess,plant,t0_.

### Statistical analysis

All analyses were carried out in R studio (version 4.2.1). The effects of watering treatment (100FC and 50FC) and mat stage (M and MD) on δ^13^C in different carbon fractions of the leaves just after labeling were assessed through mixed modelling with treatment and mat stage as fixed effects, and with table and labeling group as random effects. The effects were evaluated per leaf. The analysis was done using the *lme4* and *lmerTest* packages. To assess the effect of leaf age, data from all leaves were combined and the same mixed model was used with the addition of the fixed leaf age effect and a random plant effect. Post-hoc tests were then used to compare individual leaves (Bonferroni adjustment, *emmeans* package). The different carbon fractions were compared with paired t-tests.

Treatment and mat stage effects on relative ^13^C_excess_ in leaves, phloem sap, corm, pseudostem, roots, respiration and starch extracted from the corm were determined using simple linear models, as the random effects did not explain any variance in this response variable. The effects were assessed separately for all sampling times and per leaf group (young, active, new). At harvest, sampling group was added as a fixed effect since both sampling groups were harvested at a different time. The effect of leaf age on ^13^C_excess_ in leaves and phloem sap was assessed with paired t-tests.

In pulse labeling experiments, ^13^C_excess_ dynamics in non-labeled tissue can be described by a combined exponential logistic model (Studer et al., 2014). An initial lag phase is followed by a net import of label. ^13^C_excess_ increases until the export (or respiration) equals the import, and the maximum is reached. The initial increase can be described by a logistic model


(6) Cexcess13(%)=a1−e−k*(t−b)

where *a* is the ^13^C_excess_ at peak time, *k* the rate constant of the label influx and *b* the time of the peak. The consequent decrease in the label (when there is net export) can be described by the exponential function


(7) Cexcess13(%)=a*e−l*t+m

where *a* is the ^13^C_excess_ at peak time, *l* the rate constant of the label efflux and *m* the steady state level at which the plant part eventually remains (Studer et al., 2014). The combined model is then defined as


(8) Cexcess13(%)=a*e−l*(t−b)1−e−k*(t−b)

In this model the parameters no longer have biological meaning. We used this model to describe the change in ^13^C_excess_ in all plant parts, except for the labeled leaves of the mother plant. In those, label only decreased so the exponential function was used. Parameter estimation of the models was done through non-linear least squares regression (*nls*). Not all models were built with the same number of observations. After analyzing all samples of the optimal irrigation treatment, it was found that the observations at certain times did not contribute to the estimation of the overall trend. Those samples were not analyzed for the suboptimal watering treatment. This, however, led to a larger uncertainty in the model estimation for the suboptimal irrigation treatment. In two cases, observations at a certain time were omitted from model estimation as they did not allow the model to converge.

## Results

### Photosynthetic activity and transport in the different leaves

**Figure 2:**
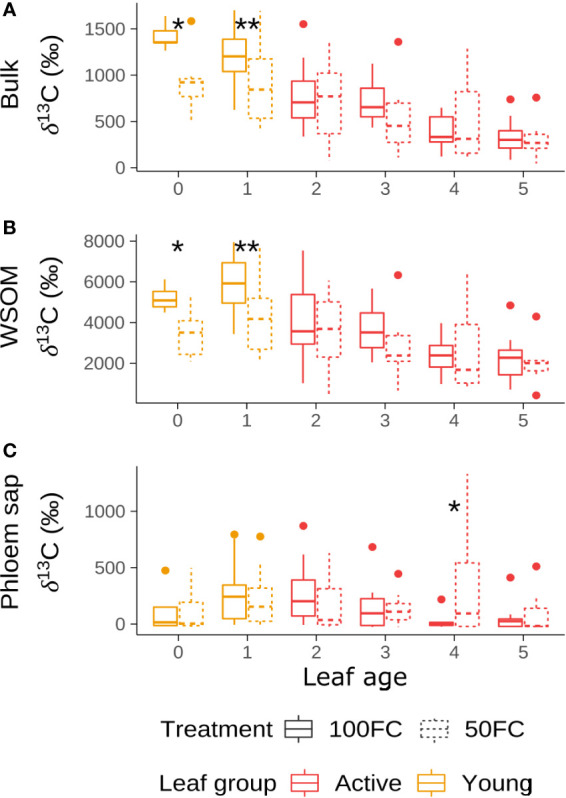
δ^13^C in all mother plant leaves just after labeling in the bulk material **(A)**, water soluble organic matter (WSOM) **(B)** and phloem sap **(C)** under optimal (100FC) and suboptimal (50FC) watering. Leaf 0 indicates the rolled up or partially opened cigar leaf, leaf 1 the 1^st^ fully opened leaf and the remaining leaves the consecutively older fully opened leaves. Significant treatment effects are indicated with * (p< 0.05) and ** (p< 0.01). Significance levels were determined through mixed modelling with labeling group and table as random effects and treatment as fixed effect.

The δ^13^C values of bulk material immediately after labeling indicated a decrease in photosynthetic activity with increasing leaf age (**Figure 2A**). The youngest leaf, leaf 0, exhibited the highest bulk δ^13^C value, suggesting the highest photosynthetic activity, while the oldest leaf, leaf 5, exhibited the lowest bulk δ^13^C value, indicating the lowest photosynthetic activity. The δ^13^C values of leaf 0 and leaf 1, the two youngest leaves, were not significantly different from each other, but were significantly higher than all active leaves (p< 0.01). Within the active leaves, there were no significant differences between consecutive leaves, but leaves that were further apart were significantly different from each other (p< 0.01).

Suboptimal watering reduced the photosynthetic activity of the young leaves. Young leaves under suboptimal watering had significantly lower δ^13^C values than young leaves of optimally watered plants (p< 0.05) (**Figure 2A**). Due to their reduced photosynthetic activity in combination with a smaller biomass, the plants subjected to suboptimal watering photosynthesized less ^13^C label overall (93 ± 11 mg) than optimally watered plants (160 ± 15 mg).

δ^13^C values in the WSOM fraction were significantly higher than in the bulk material (p< 0.01) (**Figure 2B**). As in the bulk leaf material, δ^13^C in the WSOM fraction decreased with increasing leaf age. All leaves were significantly different from each other, except for consecutive leaves and leaf 0 and leaf 2 (p< 0.05). The suboptimal irrigation treatment had the same effect on the WSOM fraction as on the bulk leaf values, namely a significant decrease in δ^13^C in both young leaves (p< 0.05).

δ^13^C values in the phloem sap differed from the values in the leaf material (**Figure 2C**). The values were overall low, indicating a limited loading of the label into the phloem, just after labeling. Moreover, there was no significant effect of leaf age as could be seen in bulk and WSOM. There was a significant treatment effect in leaf 4 (p< 0.05), with the values in the suboptimally watered plants being much higher than in optimally watered plants. This corresponds to some extent to increased values in bulk and WSOM material in those plants, but the treatment effect was not significant in those cases.

### Carbon dynamics in the phloem sap

**Figure 3:**
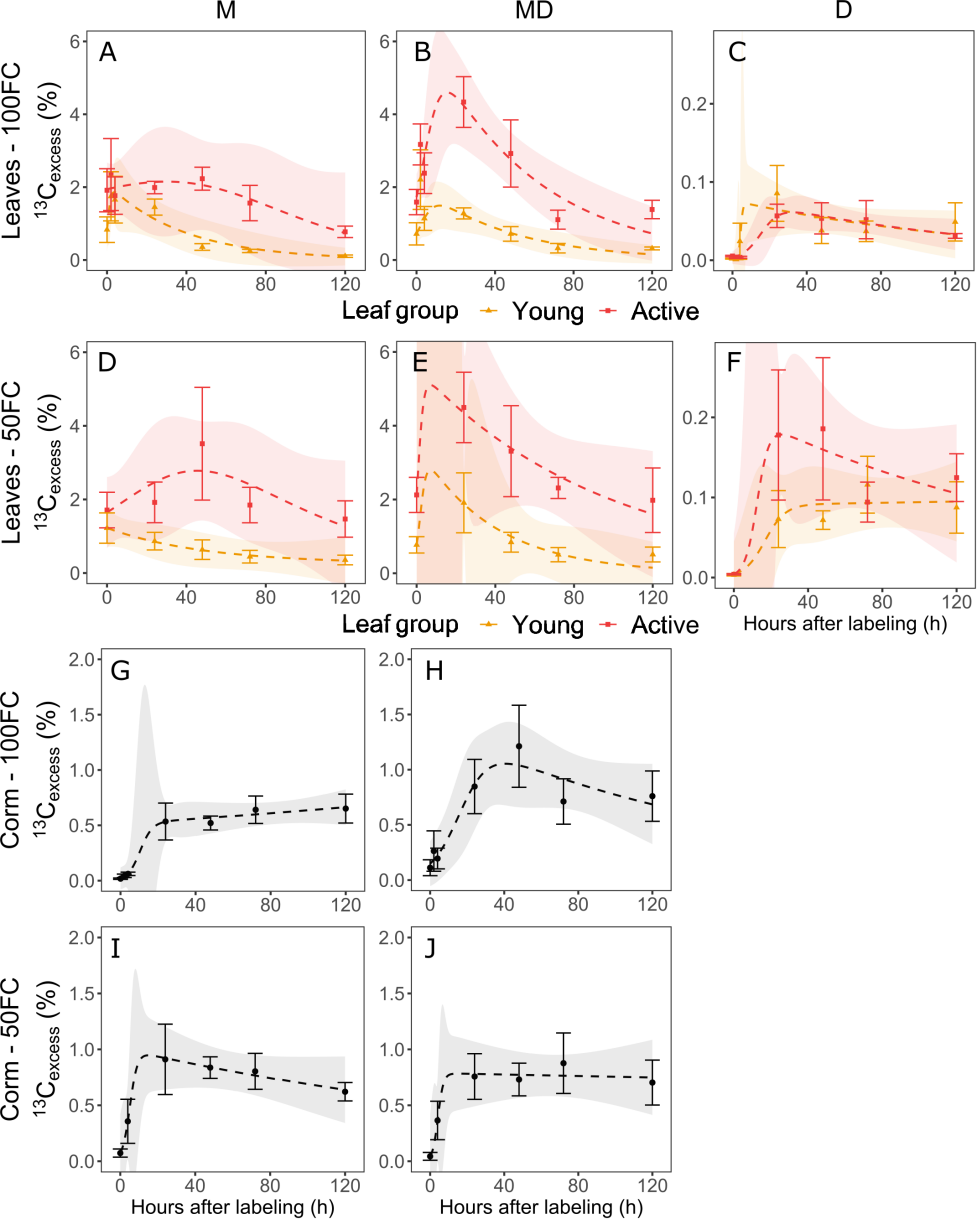
Evolution over time of ^13^C_excess_ in the phloem sap extracted from the leaf petioles in mother plants without a daughter plant (M), mother plants with a daughter plant (MD) and daughter plants (D) under optimal irrigation (100FC) **(A–C)** and suboptimal irrigation (50FC) **(D–F)** and phloem sap extracted from the corm under optimal **(G, H)** and suboptimal irrigation **(I, J)**. Error bars indicate standard error of the mean (n = 2-6) and the confidence interval of the non-linear model is given at a 95% confidence level.

The phloem sap in the leaves of the mother plants was enriched immediately after labeling, implying immediate export. In the active leaves, ^13^C_excess_ reached a maximum after 24 hours in mother plants with a daughter plant (MD) (**Figures 3B–E**) and after 48 hours in mother plants without a daughter plant (M) (**Figures 3A–D**). The maximum was significantly higher in MD plants than in M plants (p< 0.05), while after 48 hours, there was no difference between the mat stages. This implies that overall, more label was transported from the active mother plant leaves in MD plants than in M plants. In the young leaves of mother plants, ^13^C_excess_ peaked around or before 24 hours after labeling. Enrichment was overall lower than in active leaves. The difference between young and active leaves was significant from 24 hours onwards (p< 0.05). Suboptimal watering appeared to lead to increased levels in the phloem sap in both young and active leaves, but variation was large and the treatment effect was only significant in the active leaves after 72 hours (p< 0.05).

The phloem sap in the daughter plant leaves exhibited increased enrichment levels 24 hours after labeling (**Figures 3C–F**). In most leaves, the maximum enrichment was also reached at this time. Active and young leaves had similar ^13^C_excess_ levels under optimal watering conditions, whereas under suboptimal watering, higher values were observed in active leaves. The leaf age effect was however only significant 48 hours after labeling (p< 0.05). Suboptimal watering resulted in increased ^13^C_excess_ values. In active leaves, the difference was significant 120 hours after labeling and in young leaves, after 72 hours (p< 0.05).

Small amounts of the label could be detected in the corm phloem sap of a few plants right after labeling, but ^13^C_excess_ strongly increased in all plants from 24 hours after labeling (**Figures 3G–J**). After reaching a plateau, the ^13^C_excess_ levels gradually decreased, except in M plants under optimal watering, which continued to increase. MD plants under suboptimal watering had the highest maximum ^13^C_excess_ values, resulting in a significant mat stage and interaction effect 48 hours after labeling (p< 0.05).

The combined exponential logistical models described the increase and decrease in ^13^C_excess_ in the phloem sap well, but uncertainty was higher when there were fewer observations around the time of peak enrichment (**Figure 3**). Nonetheless, the overall trend coincided with the averages at each sampling time and parameter estimates of the model were generally significant (**Supplementary Table 2**).

### Carbon dynamics in bulk leaves and corm

**Figure 4:**
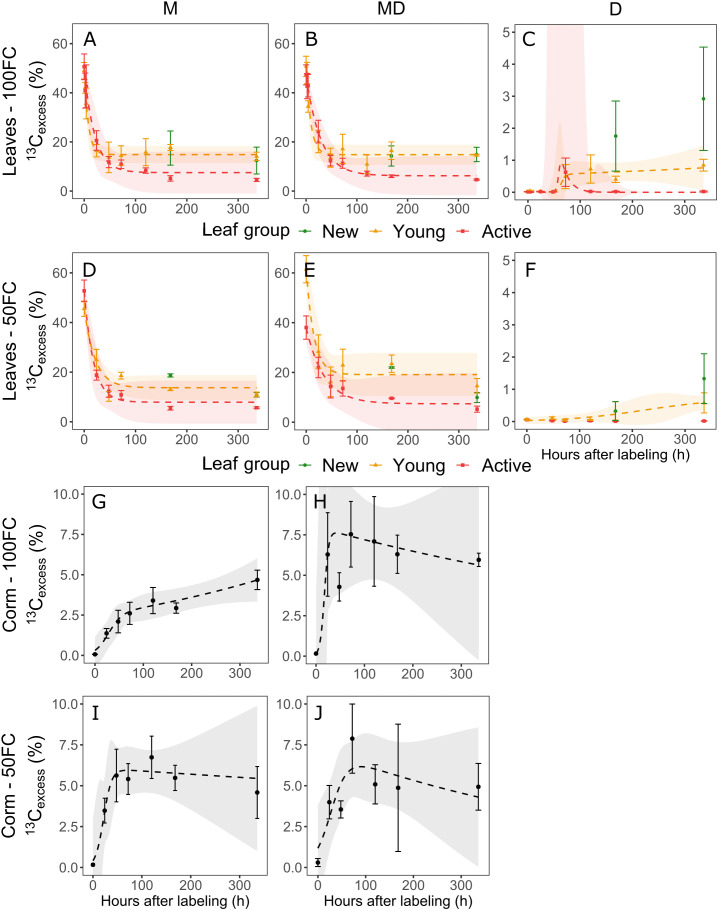
Evolution over time of ^13^C_excess_ in the bulk material of the leaves of mother plants without a daughter plant (M), mother plants with a daughter plant (MD) and daughter plants (D) under optimal irrigation (100FC) **(A–C)** and suboptimal irrigation (50FC) **(D–F)** and in the bulk material of the corm under optimal **(G,H)** and suboptimal irrigation **(I, J)**. Error bars indicate standard error of the mean (n = 2-6) and the confidence interval of the non-linear model is given at a 95% confidence level.

^13^C_excess_ in the bulk leaves of the labeled mother plants strongly decreased immediately after labeling (**Figures 4A–E**). Within 24 hours, more than 50% of the label was gone. The decrease in label appeared slightly faster in the young leaves compared to the active leaves, but the young leaves remained significantly more enriched than the active leaves (p< 0.05). Among the young leaves, leaf 0 remained more enriched than leaf 1 (data not shown). New leaves, which appeared later, had enrichment levels similar to those of young leaves and significantly higher than those of active leaves (p< 0.01). MD plants under suboptimal watering differed from the other plants in the sense that initially, most of the label was taken up by the young leaves (**Figure 4E**), while in other plants an equal amount of label was taken up by young and active leaves. There were no significant treatment effects.

Label could be detected in the daughter plant leaves after 48 hours, although only in very small amounts in the young leaves of the suboptimally watered plants (**Figure 4F**). This resulted in a significant treatment effect (p< 0.1). After that, ^13^C_excess_ in the young leaves under suboptimal watering increased gradually, but always remained below 1%. Active leaves under suboptimal watering never became enriched. Both active and young leaves in optimally watered daughter plants became enriched as of 72 hours after labeling (**Figure 4C**). ^13^C_excess_ in the active leaves immediately peaked, while young leaves maintained a constant ^13^C_excess_ level of around 1%. New leaves that appeared during the experiment where enriched as well and their enrichment kept increasing between 168 and 336 hours after labeling. In both treatments, ^13^C_excess_ values in new and young leaves were significantly higher than in active leaves after 168 and 336 hours (p< 0.05).

The corm became enriched very soon after labeling (**Figures 4G–J**). Corms under both treatments and mat stages were enriched after 24 hours, although levels varied considerably. MD plants had significantly higher ^13^C_excess_ values after 24 hours (p< 0.05), but there was also a significant interaction effect of mat stage and treatment (p< 0.1). All plants reached a maximum between 24 and 120 hours after labeling, except the M plants under optimal watering, which kept increasing. This corresponds to the continued increase in the corm phloem sap in optimally watered M plants. MD plants exhibited a larger variation in ^13^C_excess_ than M plants.

Parameter estimates of the non-linear models describing the decrease in ^13^C_excess_ in the mother plants were mostly significant, resulting in a small uncertainty (**Figure 4** and **Supplementary Table 2**). In daughter plants however, the models were highly uncertain, due to the lack of a peak or the peak being narrow. No model was estimated for the active leaves in the suboptimally watered daughter plants, as there was no change in ^13^C_excess_. In the corm, increase in the label was described well by the model, but its plateau led to a large uncertainty as the model assumes that ^13^C_excess_ decreases.

### Final carbon allocation

**Figure 5:**
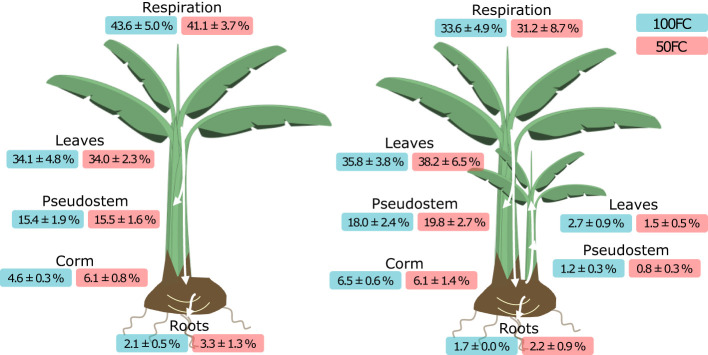
Average ^13^C_excess_ (%) and standard error of the average in all plant parts at harvest (after 168 and 336 hours) in plants without daughter plants (left) and with daughter plants (right) under optimal (100FC) and drought (50FC) treatment (n = 6). Plant design by Freepik.

After one to two weeks, most of the label was still present in the leaves of the mother plants (35.6 ± 2.2) or had been lost through respiration (37.5 ± 3.0) (**Figure 5**). Optimally watered plants without a daughter plant respired the most. Generally, suboptimal watering and the presence of a sucker appeared to result in less respiration losses, but variability among the plants was large. The interaction effect of treatment and mat stage was significant (p< 0.1). Suboptimally watered plants with a sucker retained on average most label in the leaves of the mother plant, but the effects of treatment and mat stage were not significant.

A large portion of the label was detected in the pseudostem of the mother plant (17.2 ± 1.1). This amount was significantly larger in plants with a daughter plant than in plants without a daughter plant (p< 0.1). On average, 5.8 ± 0.4% was allocated to the corm. The amount varied between the treatments and plants with/without daughter plant and was on average lowest in optimally watered plants without a daughter plant. There were however no significant effects on allocation to the corm. Allocation to the roots did not differ between treatments and mat stages.

In plants with a daughter plant, 3.1 ± 0.7% of the label eventually ended up in the daughter plant. Most of the labeled carbon was recovered in the leaves, with the highest amounts in optimally watered plants. The treatment effect on ^13^C_excess_ in the daughter plant leaves was however not significant.

It should be noted that due to the higher label uptake by the optimally watered plants compared to the suboptimally watered plants, the absolute amount of label in plants under optimal watering was much higher than in suboptimally watered plants. Hence, a similar relative allocation (%) can still imply very different absolute amounts (mg) of ^13^C_excess_. For example, mother plant’s leaves had similar relative ^13^C_excess_ in both treatments, but in absolute amounts, MD mother plants under optimal watering retained 60.6 ± 5.4 mg of excess ^13^C, while suboptimally watered MD mother plants only retained half of that, 30.0 ± 6.1 mg. All absolute amounts of ^13^C_excess_ in the different plant parts can be found in **Supplementary Table 3**.

### Enrichment of different carbon fractions in the leaves

**Figure 6:**
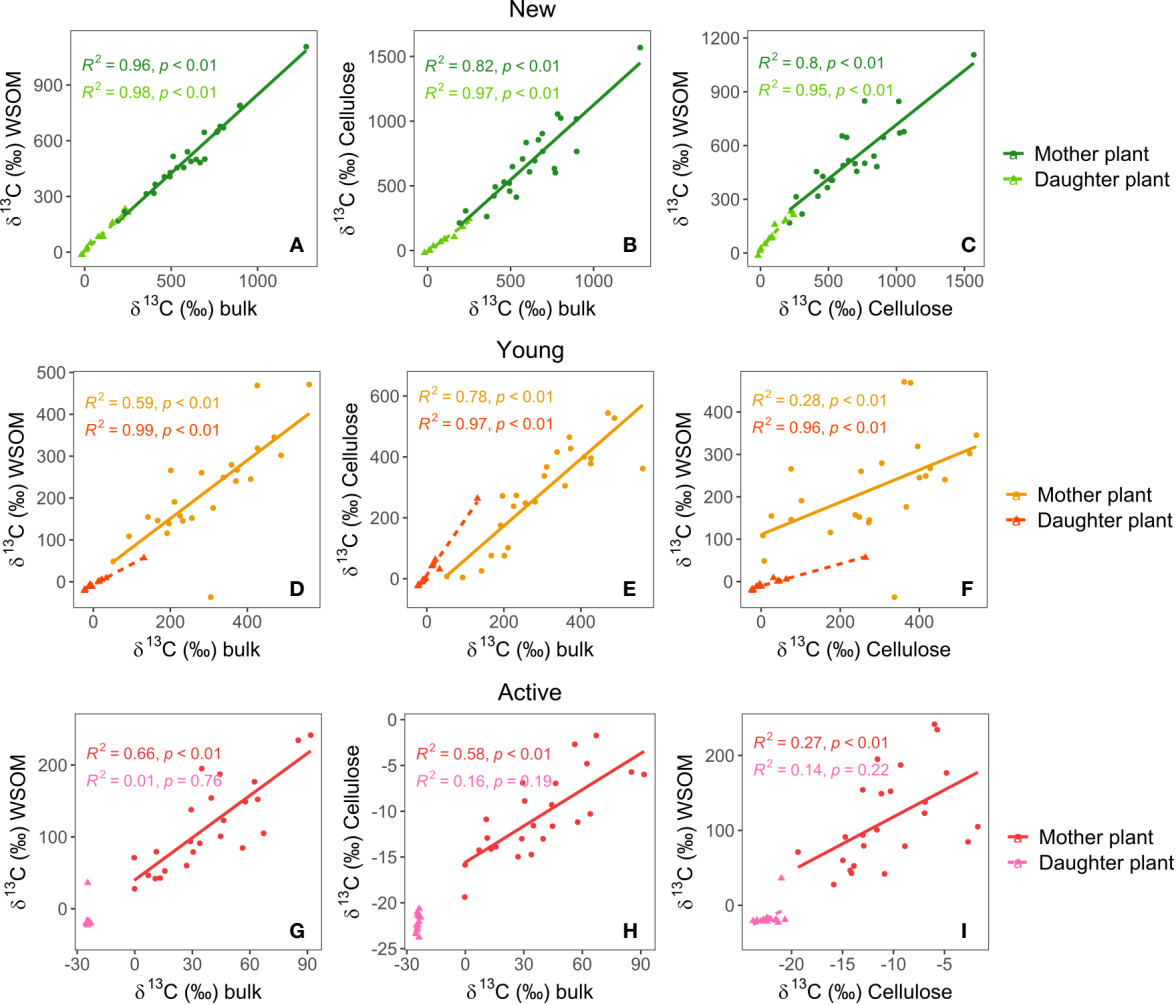
Relations between δ^13^C values in the bulk, water-soluble organic matter (WSOM) and cellulose fractions of the new **(A–C)**, young **(D–F)** and active **(G–I)** leaves of mother- and daughter plant at harvest (1 and 2 weeks after labeling).

New leaves, which developed during the experiment, had the highest enrichment levels of all leaves (**Figures 6A–C**). These leaves had the highest label uptake relative to their biomass. Bulk, WSOM and cellulose δ^13^C values in the new leaves were highly correlated, especially in the daughter plants. Bulk and WSOM δ^13^C values in mother plants were similar to each other, while cellulose δ^13^C values were significantly higher, on average about 150‰ (p< 0.01). All carbon pools had similar δ^13^C levels in daughter plants.

The fully grown active leaves had the lowest δ^13^C values (**Figures 6G–I**) and young leaves, which were partly developed, had enrichment levels in between new and active leaves (**Figures 6D–F**). In the young leaves of the mother plants, the WSOM fraction was significantly less enriched than the bulk fraction (p< 0.01). Bulk and cellulose δ^13^C had a 1:1 relation and were not significantly different. Although still high, the correlation between the δ^13^C values in the different fractions was overall lower in young leaves than in new leaves. In the daughter plants, the WSOM values were much lower than the bulk values, but cellulose δ^13^C was higher than bulk δ^13^C (p< 0.1). δ^13^C values in daughter plants were highly correlated.

In the active leaves of the mother plant finally, the WSOM fraction was the most enriched. Very little label was present in the cellulose, but correlations between the δ^13^C values in the different fractions were still high and significant. The active leaves in the daughter plants were not enriched and there were no significant correlations between the δ^13^C values of the different fractions.

### Long-term storage and variability in the corm

**Figure 7:**
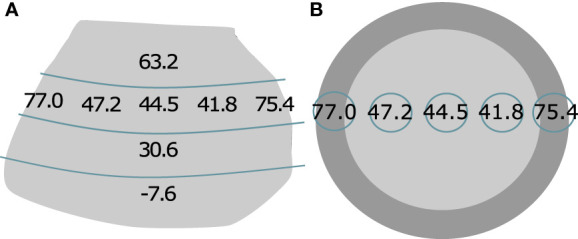
Variability in δ^13^C (‰) in a banana corm harvested one week after labeling, shown as a vertical **(A)** and horizontal **(B)** cross-section. The dark region in the horizontal cross-section indicates the cortex. Thin blue lines on both Figures indicate where the corm was cut.

The banana corm is an ill-understood plant organ. To elucidate its role in carbon dynamics, we performed several small tests. **Figure 7** shows the variability in δ^13^C measured in a single corm. We found a radial gradient in δ^13^C, whereby the enrichment was higher in the cortex and lower in the central cylinder. Within the central cylinder, enrichment did not vary horizontally. Vertically however, there was a clear gradient whereby δ^13^C values were higher, the higher up the corm and lowest in the deepest parts of the corm.

Around six percent of all label was allocated to the corm after 1-2 weeks (**Figure 5**), but less than ten percent of that was present in the form of starch (**Table 2**). To some extent, the trends in the bulk and starch material of the corm were similar. ^13^C_excess_ was the highest in optimally watered MD plants in both cases. However, while ^13^C_excess_ values of the bulk material in the corm in suboptimally watered MD plants were also fairly high, the ^13^C_excess_ values in the starch material of these plants were the lowest of all plants. ^13^C_excess_ in the corm starch in suboptimally watered MD plants was 85% lower than in optimally watered MD plants. Hence, treatment (p< 0.01), mat stage (p< 0.05) and their interaction (p< 0.01) significantly affected ^13^C_excess_ in the starch extracted from the corm. Trends in overall starch content were similar to the trends in ^13^C_excess_, with the highest average starch content in optimally watered MD plants (11.7 ± 1.2%) and the lowest average starch content in suboptimally watered MD plants (7.2 ± 1.1%). Treatment (p< 0.05) and the interaction between treatment and mat stage (p< 0.1) significantly affected starch content.

**Table 2:** Average ^13^C_excess_ (%) in starch extracted from the harvested corms (1-2 weeks after labeling) under both treatments (100% field capacity (100FC) and 50% field capacity (5FC)) and in both mat stages (plants with a daughter plant (MD) and without a daughter plant (M)) (n = 6).

Treatment	Mat stage	^13^C_excess_ (%)
100FC	MD	0.48 ± 0.07
100FC	M	0.26 ± 0.02
50FC	MD	0.18 ± 0.05
50FC	M	0.38 ± 0.11

Treatment, mat stage and their interaction had a significant effect on ^13^C_excess_ as determined through by their combined linear model.

As a test for long-term storage in the corm, several sucker-free banana plants were labeled. Once they grew a daughter plant, these were analyzed. All suckers exhibited slightly increased δ^13^C levels. The level of enrichment moreover decreased over time, reaching near natural abundance levels. The first four suckers that appeared within three months after the experiment, had an average enrichment of -15.8 ± 2.1 ‰, while the last two, which appeared after more than three months had an average enrichment of -20.8 ± 0.4 ‰.

## Discussion

### The effect of drought and sucker presence on carbon allocation in the banana mat

Plants assimilate carbon through photosynthesis. This carbon can be used to maintain plant functioning or for growth. Leaves are the major source of photo-assimilates. When there is an excess amount of carbohydrates, it is exported from the source to the sinks (Schulze et al., 2018). Banana suckers are unusual in the sense that they are photosynthetically active and thus theoretically self-supporting, but still partly rely on photo-assimilates from the mother plant (Eckstein et al., 1995; Eckstein and Robinson, 1999; Dens et al., 2008; Mahdi et al., 2014; Dorel et al., 2016). By labeling mother plants with ^13^CO_2_, we were able to determine that in Grand Nain, 3.1 ± 0.7% of the carbon fixed by the mother plant was allocated to the photosynthetically active daughter plant after one to two weeks (**Figure 5**). At that time, ^13^C_excess_ in the new leaves of the sucker was still increasing, which means the total amount might eventually be higher. If we assume the sucker has one fourth of the leaf area of the mother plant (which was more or less the case in our experiment) and consider otherwise equally efficient photosynthesis, this would mean that on average 10% of the carbon in a daughter plant originates from the mother plant. The relative allocation to the sucker was lower in suboptimally watered plants than in optimally watered plants, but the difference was not significant, likely due to the small sample size. Banana mats seem to prioritize the main plant over the vegetative reproduction organ during drought stress.

Furthermore, the presence of a sucker resulted in more carbohydrates being allocated to the pseudostem of the mother plant (**Figure 5**). The pseudostem is structurally important to carry a bunch, hence carbon allocation to the pseudostem increases just before flowering (Eckstein et al., 1995). Dens et al. (2008) however showed that the pseudostem is also an important source of carbohydrates for the sucker once the mother plant dies. It seems that the presence of a sucker initiated additional allocation to the pseudostem as storage for future growth of the sucker and possibly, a flower and bunch, irrespective of the age of the plant, as all plants in the experiment were of the same age. An important note to make is that the pseudostem is also a photosynthetically active plant part. Bulk pseudostem δ^13^C was not determined just after labeling and hence, the contribution of the pseudostem to the total label uptake has not been considered.

Carbon allocation is a trade-off. If plants with suckers allocate carbon to the sucker and pseudostem, it automatically implies less allocation to other plant parts. We hypothesized that plants without daughter plants would be able to invest more in their own growth and thus allocate more resources to the newly developing leaves of the mother plant. However, this was not the case. New leaves in mother plants with and without suckers had similar ^13^C_excess_ levels (**Figure 4**). Moreover, the phloem sap of plants without suckers showed that they exported less photo-assimilates and had higher respiratory losses, especially under optimal irrigation (**Figures 3**–**5**). Rather than diverting carbohydrates to alternative sinks, plants without suckers conserved and later respired more carbohydrates in the source leaves. This implies that plants with fewer sinks operate less efficiently. Turner (1972) also observed that when suckers were removed from the mother plant under suboptimal conditions, dry matter production dropped. He posited that the efficiency of the plant to assimilate carbon is partly determined by the internal demand. Other genetic and molecular research on, among others, fruit trees confirmed that sink removal can lead to reduced photosynthetic activity and the accumulation of sugar and other carbon compounds in the source leaves (Adams et al., 2018).

Finally, drought stress and sucker presence both increased carbon allocation to the corm. This was visible in the phloem sap and bulk fraction (**Figures 3**, **4** and **Table 2**). In the starch fraction of the corm however, the effects of treatment and growth stage were different (**Table 2**). While phloem sap and the bulk material had elevated ^13^C_excess_ levels in optimally watered plants with suckers, suboptimally watered plants with suckers and suboptimally watered plants without suckers, ^13^C_excess_ in the starch fraction was only elevated in optimally watered plants with suckers and suboptimally watered plants without suckers. Suboptimally watered plants with suckers had the lowest ^13^C_excess_ of all plants, leading to a significant interaction effect between treatment and mat stage on ^13^C_excess_ in the starch. To understand this discrepancy, it is important to keep in mind that the corm has two different functions. The corm is the central point of connectivity for all leaves of mother and daughter plant and all carbon fluxes pass through it. Hence, it fulfills the role of transport organ. At the same time, the corm stores carbohydrates in the long term in the form of starch which can serve as a buffer and is thus a storage organ. From our observations in the phloem sap and bulk material we can derive that both drought stress and suckers, as well as their combination increase the transport activity in the corm. However, carbon storage in the corm in the form of starch only increases when drought stress occurs or a sucker is present, but not when those are combined. In that case, it is severely reduced. The exact same trend occurs in overall starch content of the corm. Likely, the combination of an additional sink and a decreased carbon supply result in insufficient resources for the plant to invest in long term storage. Drought stress and sucker presence are thus impacting both activities of the corm differently. Arriving to this conclusion has only been possible by analyzing starch, aside from the bulk corm material. Only considering ^13^C_excess_ in the bulk material would have led to the incorrect conclusion that the combination of drought stress and sucker presence increases carbon storage in the corm. Therefore, we highlight the importance of analyzing the appropriate carbon fraction for specific research questions.

### Carbon allocation, storage and remobilization in the corm

Our preliminary test on variability in the corm is the first time that carbon allocation within the banana corm has been studied. The trends in δ^13^C which we found, correspond to Skutch (1932) observations of its structure. The central cylinder of the corm consists of starchy parenchyma and is crisscrossed by looping vascular bundles. δ^13^C values in this part of the corm were highly similar within the same plane (**Figure 7**). The chaotic direction of these bundles seems to lead to a horizontally homogeneous distribution of photo-assimilates. We did observe a vertical gradient in δ^13^C. A likely explanation is the orthotropic or vertical growth of the corm and the presence of the apex on the top of the corm (Devos, 1984). The vertical gradient in δ^13^C confirms that more carbohydrates were allocated to the higher parts of the corm and that growth was concentrated here. Even higher values were found in the cambium region. Previous research has shown that this is where water transport is concentrated, but our results now indicate this is also where most phloem transport is and possibly, where phloem unloading occurs (Lu et al., 2002).

Despite the horizontal symmetry observed in the corm, it should be mentioned that variability in δ^13^C in the corm bulk samples was high (**Figure 4**). Even within plants, δ^13^C values often strongly varied between two sampling times, simply because samples were taken at different sides of the corm. Since the variability was especially high in plants with a sucker, we believe this is the main reason for this discrepancy. The sucker is a sink to which photo-assimilates are transported. This would cause δ^13^C to be higher on the side of the sucker than on other sides of the corm. Hence, a corm with a sucker could be asymmetric in ^13^C distribution. The corm that is portrayed in **Figure 6** is of a plant without sucker, hence this asymmetry does not show. In addition, it should be noted that some samples only consisted of cortex rather than including the cambium and central cylinder, because both the depth of the cortex and the exact dimensions of the samples varied. For future research, we recommend making sure corm samples always include the cambium region. Despite this variability, our method, of repeatedly taking sub-samples of living corms for both phloem sap and bulk analysis, proved to work well and helped us gain useful insights.

Finally, we found that suckers that developed from labeled plants months later, still had slightly elevated δ^13^C levels. This proves that photo-assimilates were stored in the corm for months. In addition, it strongly suggests that initial sucker development is controlled by a carbon supply from the corm. Instead of directly transporting sugars from the source leaves to the developing sucker, immobile carbon in the corm was thus remobilized, loaded into the phloem sap and transported to the sucker.

### A gradual transition from sink to source leaf

Banana leaves originate in the corm and remain enclosed in the pseudostem without being exposed to light, until almost fully developed. Hence, four fifths of their α-cellulose is derived heterotrophically from other leaves (Zhenyu et al., 2020). Once the leaves appear, they become photosynthetically active and self-provisionary. Soon after that, they evolve into a source of photosynthates for the rest of the plant. In our experiment, we sampled new, young and active leaves, which were the leaves still enclosed during labeling, the ones that were partly opened and the fully-grown leaves, respectively. The new leaves had the highest δ^13^C values, confirming their role as a sink (**Figure 6**). Furthermore, bulk, WSOM and cellulose δ^13^C in the new leaves were strongly correlated, demonstrating that imported carbohydrates were effectively used to make structural cellulose. The relationship between δ^13^C in WSOM and cellulose in daughter plants was close to 1:1, while in mother plants, cellulose was generally more enriched than WSOM. This could be the result of a delay in transport to the suckers compared to the new mother plant leaves. ^13^C_excess_ in the new leaves of the daughter plant also still increased between one and two weeks after labeling, which was not the case for the mother plants (**Figure 4**). Another possible explanation is that the mother plant is more photosynthetically active than the daughter plant and hence, the ^13^C in the WSOM becomes more diluted.

As expected, the active leaves were the most important source of photosynthates. The active mother plant leaves had the lowest δ^13^C levels of all mother plant leaves at harvest, despite initially taking up about 50% of the label (**Figures 4**–**6**). Most of the label that remained was present in the WSOM fraction, while there was almost no ^13^C_excess_ in the cellulose, indicating that the cellulose in the active leaves was already fully formed at the time of labeling. The active daughter plant leaves were not enriched at all at harvest, but there was a short spike in ^13^C_excess_ 72 hours after labeling (**Figure 4**). Thus, even though active leaves are fully grown and export most of their photo-assimilates, they show some sink behavior as well.

Young leaves were less enriched than new leaves, but more than active leaves (**Figure 6**). The enrichment in the young daughter plant leaves implies that young leaves not only export, but also import carbon. Hence, they act both as source and sink. Enrichment was lowest in the WSOM fraction. In the daughter plants, the difference between the fractions was particularly large. Moreover, the correlation between the different fractions in the daughter plants was as high as in new leaves and much higher than in the young mother plant leaves. In active mother plant leaves, the correlations were even lower. It seems that when ^13^C originates from translocation, it is mainly used for cellulose development. The δ^13^C values of the different fractions correlate well, since a high sink strength implies a large import in the form of WSOM which will be converted to cellulose. In the mother plants, photosynthetically derived ^13^C on the other hand, is present in both cellulose and WSOM, which are seemingly less related, leading to lower correlations between the δ^13^C values of the different fractions. This may be a result of the different sizes of initial carbon pools or dynamic enrichment of the different carbon pools i.e. that the WSOM pool in the mother plant is consistently being replenished with new photo-assimilates leading to a lower correlation.

### Photosynthetic activity in the young leaves

δ^13^C values of leaves just after labeling reflect the uptake of ^13^C label relative to the amount of ^13^C already present. Since carbon percentage and natural abundance amount of ^13^C were the same in all leaves, this value is in indicator of the photosynthetic activity of the leaves, independent of their size. We expected to find the highest δ^13^C value in the second youngest leaf, as this is known to be the photosynthetically most active leaf (Thomas and Turner, 1998). Instead, we found that leaves 0 and 1 had similar or even higher δ^13^C values than leaf 2 (**Figure 2**). Moreover, both youngest leaves combined had the same ^13^C_excess_ as the four active leaves combined (**Figure 4**). There could be different reasons for this.

Firstly, the active leaves might have already exported more label than the young leaves, during the two hours of labeling. However, δ^13^C values in the exported phloem sap of leaf 1 were similar to leaf 2, so this could only explain the high enrichment in leaf 0, which indeed did not export much. A second and more likely explanation is light. Thomas and Turner (1998) described how photosynthetic activity of leaves is determined by chlorophyl content and photosynthetic flux density. Light exposure decreases from the youngest to the oldest leaf, while chlorophyl content increases. The combination of these two factors results in maximal photosynthetic activity in the second youngest leaf. Light intensity in the growth chamber during labeling was however much lower than under natural outside conditions. When light is the limiting factor for photosynthesis, its variability might become more important in determining variability in photosynthetic activity. Indeed, Eckstein and Robinson (1995) measured similar photosynthetic activity in leaves one to five on cloudy days. This could explain why the youngest leaf in our experiment had the highest photosynthetic activity. We believe this finding should be considered in future experiments under light-limited conditions, such as in a greenhouse. Finally, it should be noted that leaf 0 and 1 behaved differently during our experiment, hence we suggest to not combine them in a single group.

### Implications

Isotopically labeling banana mother plants has provided new insights into carbon allocation in banana mats. Photosynthetically active Grand Nain daughter plants proved to still depend on mother plants, receiving around 3% of the mother plant’s photo-assimilates. This results in 10% of the carbon of the daughter plants being derived from the mother plant. Removing this sink does not result in an increase in the amount of carbon allocated to the mother plant. However, when resources are limited i.e. under drought stress, allocation to both the corm and daughter plant decreases. As much as 85% less carbon is stored in the corm in the form of starch under stressed conditions compared to optimal conditions. This implies that less carbon will be available for the flower and fruits that will develop in the future, as these are supplied by the corm. Allocation to the daughter plant decreases with 45% due to drought stress, which is less severe than in the corm. Nonetheless, this means that both the yield from the mother plant, as well as the potential yield from the next generation will be impacted by drought stress when a sucker is present. In order to sustain yields under suboptimal conditions, farmers might consider reducing the number of suckers or delaying sucker selection until conditions are more favorable.

We expected that the flux of carbohydrates from mother to daughter plant could have consequences for the interpretation of natural abundance δ^13^C values in daughter plants as a proxy for stress. However, we found that its impact is negligible. Regarding the choice of leaves for δ^13^C measurements, we recommend the use of active leaves i.e. the second to fifth fully open leaves, which is also currently common practice. Young leaves were found to partly act as carbon sinks, so both their bulk and phloem sap values could provide incorrect or confusing information. While phloem sap provides information about currently perceived conditions, bulk leaves represent the time frame from leaf origination in the corm until the leaf becomes the second fully open leaf and is fully grown. For photosynthetic measurements in light-limited conditions, it might make sense to measure the first fully opened leaf, rather than the second, as it could have a higher photosynthetic activity.

## Conclusion

Banana mats consist of a mother plant and one or more suckers that are photosynthetically active, but remain connected to the mother plant. We found that the mother plant translocated 3.1 ± 0.7% of its own recently assimilated photosynthates to the sucker. This implies that the suckers derived an estimated 10% of their carbon from the mother plant. Drought stress seemed to reduce allocation to the sucker. Sucker presence also led to increased carbon accumulation in the pseudostem, but the absence of a sucker did not lead to increased carbon allocation to the mother plant. Furthermore, we found that drought stress and sucker presence had various effects on the corm. Sucker presence, drought stress and their combination led to increased carbon transport through the corm as evidenced by increased ^13^C levels in the phloem sap and bulk fraction. However, while storage in the corm in the form of starch increased under drought stress or in the presence of a sucker, the combination of stress and sucker presence strongly decreased starch accumulation. The active leaves, i.e. the second to fifth fully open leaves were the most important source of photo-assimilates in the plant but fixed the same amount of carbon as the two younger leaves put together. The young leaves in our experiment were more photosynthetically active than reported in other experiments. We ascribe this to the low light-intensity in the growth chamber. The young leaves, which were not yet fully developed, imported, and exported carbon simultaneously. The active leaves thus remain the preferred choice for δ^13^C measurement to evaluate drought stress. In conclusion, by ^13^C labeling mother plants, we have been able to quantify carbon fluxes in banana mats which proved to be affected by drought stress and sucker presence in a similar way. Both led to increased allocation to storage tissues, but when combined, the insufficient availability of assimilates resulted in a decrease in starch accumulation in the corm and a decrease in the amount of carbon allocated to the sucker. Furthermore, the flux from mother plant to sucker has a negligible effect on the δ^13^C value of the sucker, hence δ^13^C can be used as proxy for stress in both mother plants and suckers.

## Data availability statement

The raw data supporting the conclusions of this article will be made available by the authors, without undue reservation.

## Author contributions

MV designed the experiment. MV and EB performed the experiment and subsequent laboratory analysis and analyzed the data. GD, RH-N and RM supervised the experimental design, execution and analysis. MV wrote the manuscript. GD, RH-N and RM reviewed and discussed draft versions of the manuscript. All authors contributed to the article and approved the submitted version.
