# Supplementary material for: 13C labeling unravels carbon dynamics in banana between mother plant, sucker and corm under drought stress
Source: Front Plant Sci. 2023 May 8;14:1141682. doi: 10.3389/fpls.2023.1141682 (PMC10286810; doi:10.3389/fpls.2023.1141682)
Supplement: Supplementary file 1 [file Table_1.docx]

**Supplementary Table 1.** Initial label uptake of all plants, measured in the leaves and phloem sap of the mother plant and the corm, expressed in mg and % ^13^C_excess_. Mat stage (M – mother plant without daughter, MD – mother plant with daughter) and treatment (100FC – watering to 100% field capacity, 50FC – watering to 50% field capacity) of the plant are given.

| **Plant** | **Treatment** | **Mat stage** | **Leaves** | | | | **Phloem sap** | | | | **Corm** | | **Total** |
| --- | --- | --- | --- | --- | --- | --- | --- | --- | --- | --- | --- | --- | --- |
|  |  |  | **Young** | | **Active** | | **Young** | | **Active** | | **-** | |  |
|  |  |  | **mg** | **%** | **mg** | **%** | **mg** | **%** | **mg** | **%** | **mg** | **%** | **mg** |
| 1 | 100FC | MD | 104.57 | 57.89 | 74.05 | 41.00 | 0.60 | 0.33 | 0.99 | 0.55 | 0.41 | 0.23 | 180.61 |
| 2 | 50FC | MD | 42.82 | 52.69 | 36.41 | 44.81 | 1.42 | 1.74 | 0.61 | 0.75 | 0.00 | 0.00 | 81.26 |
| 3 | 100FC | M | 115.70 | 55.48 | 88.89 | 42.63 | 1.49 | 0.72 | 2.36 | 1.13 | 0.09 | 0.04 | 208.52 |
| 4 | 50FC | M | 35.46 | 44.98 | 41.94 | 53.20 | 0.27 | 0.34 | 1.00 | 1.26 | 0.17 | 0.21 | 78.83 |
| 5 | 100FC | MD | 66.67 | 44.01 | 78.74 | 51.98 | 3.86 | 2.55 | 2.03 | 1.34 | 0.18 | 0.12 | 151.48 |
| 6 | 50FC | MD | 38.64 | 60.37 | 22.87 | 35.73 | 0.10 | 0.16 | 2.34 | 3.66 | 0.05 | 0.08 | 64.02 |
| 7 | 100FC | M | 26.23 | 53.29 | 20.46 | 41.55 | 0.25 | 0.52 | 2.23 | 4.52 | 0.06 | 0.13 | 49.23 |
| 8 | 50FC | M | 24.22 | 52.99 | 21.09 | 46.15 | 0.07 | 0.15 | 0.16 | 0.34 | 0.17 | 0.38 | 45.71 |
| 9 | 50FC | MD | 48.90 | 53.34 | 40.78 | 44.48 | 0.55 | 0.61 | 1.35 | 1.47 | 0.09 | 0.10 | 91.67 |
| 10 | 100FC | M | 43.37 | 35.96 | 76.16 | 63.13 | 0.26 | 0.21 | 0.75 | 0.63 | 0.09 | 0.07 | 120.63 |
| 11 | 50FC | MD | 13.08 | 82.59 | 2.61 | 16.51 | 0.07 | 0.43 | 0.07 | 0.47 | 0.00 | 0.00 | 15.83 |
| 12 | 100FC | M | 105.09 | 50.29 | 95.01 | 45.47 | 2.02 | 0.97 | 6.74 | 3.22 | 0.10 | 0.05 | 208.96 |
| 13 | 100FC | MD | 70.76 | 47.21 | 77.38 | 51.63 | 0.31 | 0.20 | 1.28 | 0.85 | 0.17 | 0.11 | 149.89 |
| 14 | 50FC | MD | 87.24 | 67.48 | 36.88 | 28.53 | 1.81 | 1.40 | 3.22 | 2.49 | 0.14 | 0.11 | 129.29 |
| 15 | 100FC | M | 60.80 | 50.19 | 54.33 | 44.85 | 3.59 | 2.96 | 2.33 | 1.93 | 0.08 | 0.07 | 121.14 |
| 16 | 50FC | M | 45.60 | 54.19 | 34.31 | 40.78 | 2.51 | 2.98 | 1.71 | 2.03 | 0.02 | 0.03 | 84.15 |
| 17 | 100FC | MD | 121.12 | 55.83 | 90.01 | 41.49 | 2.45 | 1.13 | 3.31 | 1.52 | 0.06 | 0.03 | 216.94 |
| 18 | 50FC | MD | 66.44 | 49.32 | 59.81 | 44.40 | 1.89 | 1.40 | 4.78 | 3.55 | 1.78 | 1.32 | 134.70 |
| 19 | 100FC | M | 100.48 | 37.83 | 162.97 | 61.35 | 0.13 | 0.05 | 1.88 | 0.71 | 0.16 | 0.06 | 265.63 |
| 20 | 50FC | M | 67.95 | 52.17 | 59.49 | 45.67 | 2.49 | 1.91 | 0.33 | 0.26 | 0.00 | 0.00 | 130.26 |
| 21 | 100FC | MD | 78.01 | 46.01 | 86.08 | 50.77 | 0.18 | 0.11 | 4.81 | 2.84 | 0.47 | 0.28 | 169.55 |
| 22 | 50FC | M | 46.24 | 32.43 | 91.72 | 64.34 | 0.19 | 0.13 | 4.40 | 3.09 | 0.01 | 0.01 | 142.56 |
| 23 | 100FC | MD | 80.12 | 50.31 | 74.58 | 46.83 | 0.97 | 0.61 | 3.36 | 2.11 | 0.22 | 0.14 | 159.25 |
| 24 | 50FC | M | 55.63 | 37.03 | 88.21 | 58.72 | 1.11 | 0.74 | 4.66 | 3.10 | 0.61 | 0.40 | 150.22 |
