# Supplementary material for: 13C labeling unravels carbon dynamics in banana between mother plant, sucker and corm under drought stress
Source: Front Plant Sci. 2023 May 8;14:1141682. doi: 10.3389/fpls.2023.1141682 (PMC10286810; doi:10.3389/fpls.2023.1141682)
Supplement: Supplementary file 2 [file Table_2.docx]

**Supplementary Table 2.** Model parameters of equation (5) and (6), describing the ^13^C_excess_ dynamics in bulk leaves and corm and phloem sap of the leaves and corm. The model was estimated for the different growth stages (mother plant, daughter plant), mat stages (M – mother plant without daughter, MD – mother plant with daughter), treatments (100FC – watering to 100% field capacity, 50FC – watering to 50% field capacity) and leaf groups (young, active). The significance of the parameter estimates was estimated with a t-test: ****≤0.001; ***≤0.01; ** ≤0.05; *≤0.1.

| **Plant part** | **Sample type** | **Growth stage** | **Mat stage** | **Treatment** | **Leaf group** | **a** | **b** | **k** | **l** | **m** |
| --- | --- | --- | --- | --- | --- | --- | --- | --- | --- | --- |
| Leaves | Bulk | Mother plant | M | 100FC | Young | 34.27**** |  |  | 0.12** | 14.84**** |
| Leaves | Bulk | Mother plant | M | 100FC | Active | 42.04**** |  |  | 0.05** | 7.53* |
| Leaves | Bulk | Mother plant | MD | 100FC | Young | 36.16**** |  |  | 0.11** | 14.82**** |
| Leaves | Bulk | Mother plant | MD | 100FC | Active | 40.87**** |  |  | 0.03*** | 6.24 |
| Leaves | Bulk | Mother plant | M | 50FC | Young | 31.88**** |  |  | 0.05*** | 13.74**** |
| Leaves | Bulk | Mother plant | M | 50FC | Active | 44.80**** |  |  | 0.05** | 7.90* |
| Leaves | Bulk | Mother plant | MD | 50FC | Young | 42.37**** |  |  | 0.07* | 19.14**** |
| Leaves | Bulk | Mother plant | MD | 50FC | Active | 30.49*** |  |  | 0.03* | 7.43 |
| Leaves | Bulk | Daughter plant | MD | 100FC | Young | 0.57** | 63.93 | 0.22 | 0.00 |  |
| Leaves | Bulk | Daughter plant | MD | 100FC | Active | 1.41 | 59.97 | 0.46 | 0.07 |  |
| Leaves | Bulk | Daughter plant | MD | 50FC | Young | 0.71 | 223.41 | 0.01 | - |  |
| Leaves | Bulk | Daughter plant | MD | 50FC | Active | - | - | - | - |  |
| Corm | Bulk | - | M | 100FC | - | 2.59**** | 26.60** | 0.07 | 0.00** |  |
| Corm† | Bulk | - | MD | 100FC | - | 7.85*** | 17.56 | 0.22 | 0.00 |  |
| Corm | Bulk | - | M | 50FC | - | 6.06**** | 21.85*** | 0.12 | 0.00 |  |
| Corm | Bulk | - | MD | 50FC | - | 6.98*** | 28.42 | 0.06 | 0.00 |  |
| Leaves | Phloem sap | Mother plant | M | 100FC | Young | 2.08*** | 0.36 | 1.12 | 0.03** |  |
| Leaves | Phloem sap | Mother plant | M | 100FC | Active | 4.02 | 52.18 | 0.03 | 0.02 |  |
| Leaves†† | Phloem sap | Mother plant | MD | 100FC | Young | 2.60**** | 5.28 | 0.21 | 0.03*** |  |
| Leaves | Phloem sap | Mother plant | MD | 100FC | Active | 5.81**** | 5.37 | 0.18 | 0.01*** |  |
| Leaves | Phloem sap | Mother plant | M | 50FC | Young | 0.97 |  |  | 0.02 | 0.25 |
| Leaves | Phloem sap | Mother plant | M | 50FC | Active | 5.29* | 31.65 | 0.05 | 0.02 |  |
| Leaves | Phloem sap | Mother plant | MD | 50FC | Young | 2.56**** | 2.23 | 0.33 | 0.02** |  |
| Leaves | Phloem sap | Mother plant | MD | 50FC | Active | 5.76**** | 4.03 | 0.20 | 0.01*** |  |
| Leaves | Phloem sap | Daughter plant | MD | 100FC | Young | 0.07*** | 4.45* | 1.60 | 0.01 |  |
| Leaves | Phloem sap | Daughter plant | MD | 100FC | Active | 0.07**** | 15.98** | 0.20** | 0.01 |  |
| Leaves | Phloem sap | Daughter plant | MD | 50FC | Young | 0.09** | 16.65 | 0.18 | 0.00 |  |
| Leaves | Phloem sap | Daughter plant | MD | 50FC | Active | 0.20*** | 12.81 | 0.31 | 0.01 |  |
| Corm | Phloem sap | - | M | 100FC | - | 0.52**** | 10.31 | -0.31 | -0.31 |  |
| Corm | Phloem sap | - | MD | 100FC | - | 1.27*** | 16.91* | 0.13** | 0.01 |  |
| Corm | Phloem sap | - | M | 50FC | - | 0.99**** | 5.18** | -0.49 | -0.49 |  |
| Corm | Phloem sap | - | MD | 50FC | - | 0.79**** | 4.22** | 0.67 | 0.00 |  |
| †Observations from 48 hours after labeling were excluded as their inclusion did not allow the model to converge. | | | | | | | | | |  |
| ††Observations from 2 hours after labeling were excluded as their inclusion did not allow the model to converge. | | | | | | | | | |  |
