# Supplementary material for: 13C labeling unravels carbon dynamics in banana between mother plant, sucker and corm under drought stress
Source: Front Plant Sci. 2023 May 8;14:1141682. doi: 10.3389/fpls.2023.1141682 (PMC10286810; doi:10.3389/fpls.2023.1141682)
Supplement: Supplementary file 3 [file Table_3.docx]

**Supplementary table 3.** Excess ^13^C (mg) in the different plant parts at harvest (1-2 weeks after labeling) in plants with (MD) and without (M) daughter plant, under optimal (100FC – watering to 100% field capacity) and suboptimal (50FC – watering to 50% field capacity) watering.

| **Plant part** | **M - 100FC** | **MD - 100FC** | **M - 50FC** | **MD - 50FC** |
| --- | --- | --- | --- | --- |
| Mother plant leaves | 53.2 ± 12.5 | 60.6 ± 5.4 | 35.9 ± 5.9 | 30.0 ± 6.0 |
| Mother plant petioles | 25.4 ± 6.1 | 30.7 ± 4.0 | 15.5 ± 2.3 | 17.2 ± 3.8 |
| Corm | 7.7 ± 1.8 | 10.8 ± 0.8 | 6.2 ± 1.2 | 4.7 ± 1.2 |
| Daughter plant leaves |  | 4.5 ± 1.4 |  | 1.2 ± 0.7 |
| Daughter plant petioles |  | 2.0 ± 0.4 |  | 0.4 ± 0.1 |
| Respiration | 72.3 ± 17.0 | 58.8 ± 10.7 | 44.9 ± 9.6 | 30.5 ± 12.8 |
